# Self-aligned single-electrode actuation of tangential and wineglass modes using PMN-PT

**DOI:** 10.1038/s41378-023-00521-3

**Published:** 2023-05-04

**Authors:** Ozan Erturk, Kilian Shambaugh, Ha-Seong Park, Sang-Goo Lee, Sunil A. Bhave

**Affiliations:** 1 OxideMEMS Lab, Purdue University, West Lafayette, IN USA; 2 Polytec Inc., Irvine, CA USA; 3 iBULe Photonics Company Ltd., Incheon, 21999 South Korea

**Keywords:** Sensors, Electrical and electronic engineering

## Abstract

Considering the evolution of rotation sensing and timing applications realized in micro-electro-mechanical systems (MEMS), flexural mode resonant shapes are outperformed by bulk acoustic wave (BAW) counterparts by achieving higher frequencies with both electrostatic and piezoelectric transduction. Within the 1–30 MHz range, which hosts BAW gyroscopes and timing references, piezoelectric and electrostatic MEMS have similar transduction efficiency. Although, when designed intelligently, electrostatic transduction allows self-alignment between electrodes and the resonator for various BAW modes, misalignment is inevitable regarding piezoelectric transduction of BAW modes that require electrode patterning. In this paper transverse piezoelectric actuation of [011] oriented single crystal lead magnesium niobate–lead titanate (PMN–PT) thin film disks are shown to excite the tangential mode and family of elliptical compound resonant modes, utilizing a self-aligned and unpatterned electrode that spans the entire disk surface. The resonant mode coupling is achieved by employing a unique property of [011] PMN–PT, where the in-plane piezoelectric coefficients have opposite signs. Fabricating 1-port disk transducers, RF reflection measurements are performed that demonstrate the compound mode family shapes in the 1–30 MHz range. Independent verification of mode transduction is achieved using in-plane displacement measurements with Polytec’s laser Doppler vibrometer (LDV). While the tangential mode achieves a 40^o^/s dithering rate at 335 kHz resonant frequency, the *n* = 2 wine-glass mode achieves 11.46 nm tip displacement at 8.42 MHz resonant frequency on a radius of 60 μm disk resonator in air. A single electrode resonator that can excite both tangential and wine-glass modes with such metrics lays the foundation for a BAW MEMS gyroscope with a built-in primary calibration stage.

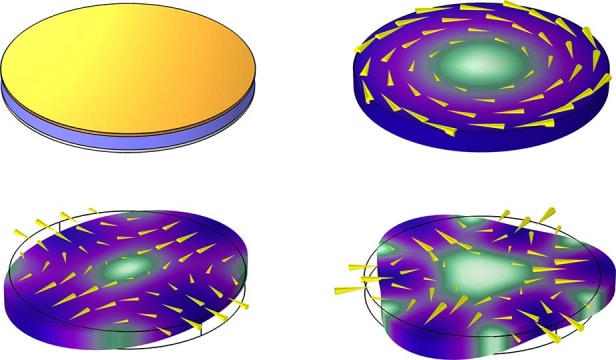

## Introduction

Contour-mode bulk acoustic wave resonators have gained attention in the past decade due to their ability to beat the limitations of flexural mode resonators (such as low *Q* and low resonance frequency) for various application domains from RF filters to gyroscopes. Considering disk structures, displacement profiles of all contour vibrations of circular plates have been reported^1^ (a correction to frequency equation to *n* = 2 wine-glass mode is published in a later work^2^), where modes of vibrations are classified into two parts when circumferential order *n* is equal to 0: namely radial mode (absence of rotation) and tangential mode (absence of areal dilation). The higher-order in-plane compound modes (*n* > 1) incorporate both tangential and areal dilation at the same time. While compound modes (especially *n* = 2 and *n* = 3) are commonly used as rotation sensors (gyroscopes)^3,4^, the tangential mode is isochoric with no out-of-plane-displacement, which is an attractive feature for MEMS clocks, resonant sensors, and an integrated calibration stage for gyroscopes.

**Fig. 1:**
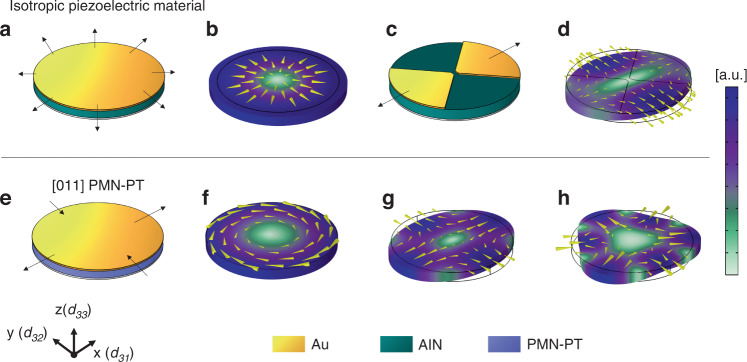
Schematic representation of disk resonator with single or split electrodes along with mode shapes of interest for conventional piezoMEMS materials (top row) and for PMN–PT (bottom row) Quiver plots (yellow cones that are proportional to the displacement magnitude) and color scale show displacement of each mode. **a** Disk resonator employing conventional piezoelectric material such as AlN with an unpatterned top electrode able to excite only radial mode due to in-plane strain having the same direction. **b** Corresponding radial mode with exaggerated deformation (due to in-plane piezoelectric coefficients (*d*_31_ and *d*_32_) having the same sign. **c** Disk resonator with segmented electrodes using the same in-plane piezoelectric coefficients with the same sign in order to excite *n* = 2 wine-glass mode with strain pointing outward only on the segmented electrodes. **d** The exaggerated deformation of strain profiles in *x*- and *y*-direction corresponding to wine-glass mode shape excited due to segmented electrode configuration. **e** Disk resonator employing [011] PMN–PT with a single electrode that is capable of exciting all the following mode shapes without the need for segmented electrodes due to in-plane piezoelectric coefficients with opposite sign, hence enforcing in-plane strain that is in opposite direction. **f** Volume conserving tangential mode shape showing shear displacement. **g** *n* = 2 wine-glass mode, where the maximum displacement point is along *x*-axis. **h** Higher order *n* = 3 wine-glass mode shape

In conventional piezoelectric MEMS materials such as aluminum nitride (AlN), or lead zirconium titanate (PZT), when a disk resonator is designed with unpatterned electrodes as shown in Fig. 1a, it is possible to excite radial modes, but not *n* = 2 wineglass mode, due to the charge cancellation occurs as a result of the strain direction being opposite in each cycle. The opposite strain direction with the same sign coupling causes the net charge to be practically zero when summed over the entire top surface; thus, a segmented electrode configuration such as shown in Fig. 1c is necessary to avoid charge cancellation^1,5–7^. Figure 1c shows a schematic representation of such segmented electrode configuration that is necessary to transduce *n* = 2 compound mode (wine-glass mode) as an example. The family of compound modes (*n* > 1) has been demonstrated to be useful in RF filters^8,9^ and gyroscopes^3,4,10^. However, the use of segmented electrodes for mode shapes of importance such as wine-glass modes inevitably causes misalignment in piezoelectric transduction topology due to the patterning of top electrodes that require a separate lithographic step during fabrication. Misalignment of the electrodes causes excitation of spurious modes that are in close proximity to the mode of interest in the frequency domain or cause undesired out-of-plane components^8^.

Self-alignment is possible in the electrostatic actuation of compound modes since the same structural layer is used to realize electrodes and the resonant body^10,11^. It is not possible to achieve self-alignment in the piezoelectric domain to realize any resonant mode other than the radial mode depicted in Fig. 1b. Therefore, even if piezoelectric transduction is accepted to provide more robust integration and efficient coupling compared to electrostatic transduction of BAW modes, in recent works it has been reported that microfabrication advancements allowed the comparable performance of BAW gyroscopes and timing references for electrostatically actuated BAW modes^10,12–15^. As electrostatic transduction is achieved through normal forces, the shear displacement required to achieve tangential mode is not possible to excite using electrostatics. Moreover, the tangential mode cannot be excited with traditional piezoelectric materials because their in-plane piezoelectric coefficients have the same sign^1,7,16^. Therefore, there has been no demonstration of a tangential mode or compound mode using a single unpatterned electrode.

In this paper, we demonstrate the efficient transduction of compound modes as well as the tangential mode in a piezoelectric disk resonator by a single self-aligned electrode utilizing the in-plane anisotropy of [011] lead magnesium niobate–lead titanate (PMN–PT). The novelty of this work establishes the realization of tangential modes and compound modes using a single electrode that was reported to be not possible in disk geometry. Utilization of a single electrode allows a self-alignment feature providing a robust and misalignment-free device fabrication. We extensively report on the transduction of *n* = 2 wine-glass mode (WGM) because it is of particular interest for gyroscopy. In addition, we measure and optically verify the tangential mode transduction.

### Lead magnesium niobate–lead titanate (PMN–PT)

PMN–PT is a relaxor-type ferroelectric material, discovered in 1997. It quickly became popular in various applications due to its superior piezoelectric constant and high coupling coefficient values as well as its ability to be grown in single crystal form^17,18^. [011]_c_ single crystal PMN–PT is reported to have opposite polarity of the in-plane piezoelectric coefficients^19^, namely *d*_31_ and *d*_32_ as shown in Fig. 1. This unique property is utilized to rotate magnetic domains in multiferroic structures, taking advantage of the compressive stress along one direction and tensile stress along the orthogonal in-plane direction^20,21^. Correspondingly, the WGM shape is realized without the need for segmented electrodes since PMN–PT produces in-plane strain that has opposite direction in *x*- and *y*-axes (as shown in Fig. 1e) under transverse electric field, hence enforcing wine-glass mode shape naturally. Although not as intuitive, the unpatterned electrode can excite the tangential mode on the same disk structure (as depicted in Fig. 1f) as well as higher order *n* = 3 WGM shape (Fig. 1h). Excitation of *n* = 3 wine glass mode shape may not be the most efficient way to couple with a single full electrode as some level of charge cancellation may reduce the coupling efficiency. Nonetheless, it is a byproduct of the fact that the anisotropy of the in-plane piezoelectric coupling coefficients is sufficient to avoid charge cancellation on the full electrode for this strain profile as it is for the *n* = 2 wine-glass mode. In a piezoelectric disk with *d*_31_ and *d*_32_ having the same sign, the net charge produced by axially symmetric points on the disk would cancel out when in-plane rotation around the *z*-axis is considered. Therefore, the tangential mode cannot be electrically excited unless there exists opposite direction strain on orthogonal axes in the plane of contour motion.

## Results

### Device design and characterization

**Fig. 2:**
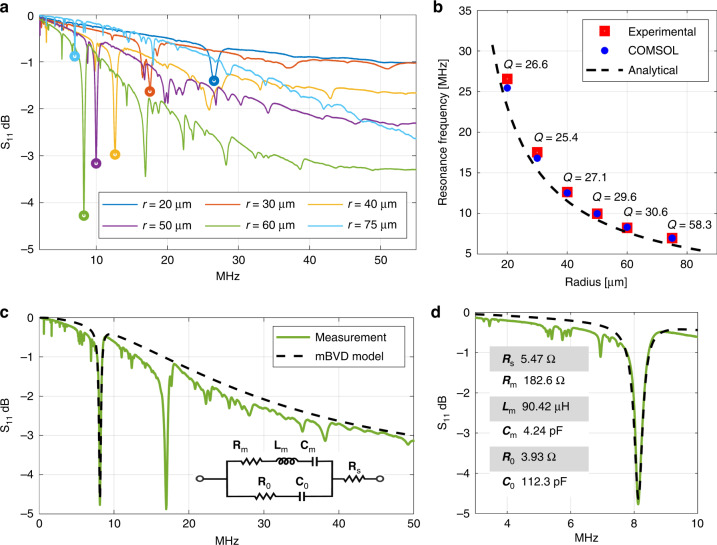
Electrical characterization results of disk resonators with varying radii with corresponding resonant frequencies. **a** Overlaid plots of measured *S*_11_ data of disks with varying radii. The resonant dip that corresponds to WGM is encircled with the same line color of the corresponding device. An explanation on the second dip around the n = 2 WGM for the *r* = 30 μ*m* device is provided in Supplementary Note 5. **b** WGM frequency vs. disk radius, analytical, FEA, and measurement results compared, and the corresponding Q-factor value of each WGM is presented. Note that the effect of anchors and tethers gets more drastic as the disk radius gets relatively smaller, hence the empirical results deviate more from the calculated values, especially for resonant frequency values estimated by analytical equations. **c** *S*_11_ data overlaid with the mBVD model of the *r* = 60 μm device. Circuit schematic of the mBVD model is provided in the inset. **d** Closeup of the WGM *S*_11_ data shown in **c** along with the corresponding lumped element values of the mBVD model

In order to investigate the transverse actuation of modes of interest and their operation of frequency, finite element analysis (FEA) modeling of the disk resonators is performed using the COMSOL piezoelectric module. Adopting the material properties of [011] PMN-PT with *d*_31_ and *d*_32_ opposite signs^19^, a simplified COMSOL model with side tethers is implemented. This model calculates the mode shapes as shown in Fig. 1f–h, and resonant frequencies of disk resonators with various radii ranging from 20 to 75 μm, where resonant frequencies as a function of disk radius are plotted in Fig. 2b.

Considering the most important geometric factor that determines the resonant frequency, namely the radius of the disk, we designed disk resonators that have resonance frequency around 10 MHz for WGM with radii (*r*_d_ in Fig. 5g) ranging from 20 to 75 μm. Given the material properties and analytical equations provided^1^ (a correction term of 2 to the *n* = 2 wine-glass mode frequency equations is provided^2^), the estimation of radius values necessary to obtain WGM in the 1–30 MHz is determined and verified by FEA analysis. Side tethers are designed to decrease mechanical loss by choosing the lengths of the tethers (*l*_t_ in Fig. 5g) to match the quarter wavelength of *n* = 2 WGM of varying radii. The width of the tethers (*w*_t_ in Fig. 5g) is chosen to be as narrow as possible to mitigate anchor losses^2,22^. Thus, the tether width is defined as 5 μm while the width of the metal trace (3 μm) is bound by the smallest width produced by the direct write lithographic tool in a repeatable fashion for the lift-off process.

Electrical RF reflection (*S*_11_) measurements are performed on fabricated 1-port devices in air after the release step using ground-signal-ground (GSG) pads. Figure 2a shows the RF response of disk resonators with radii values ranging from 20 to 75 μm. It is shown that although spurious modes exist for some radii values, the most prominent resonant shape in the range of interest is the desired WGM. In Fig. 2c and d, the modified Butterworth–Van Dyke (mBVD) model along with the circuit model parameters of the *r* = 60 μm device is provided. A detailed explanation of the model is provided in Supplementary Note 3.

**Fig. 3:**
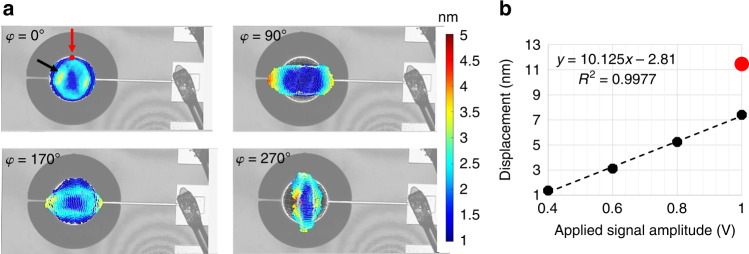
LDV results for *n* = 2 wine-glass mode. **a** LDV snapshots of a disk resonator with *r* = 60 μm radius shown at different phases of a single oscillation cycle at *φ* = 0°, *φ* = 90°, *φ* = 170°, and *φ* = 270°. **b** Displacement in the *y*-direction as a function of applied signal amplitude. Two distinct data points are plotted while the maximum tip location is recorded (noted in the red circle in the *φ* = 0° phase) while an inner point (noted by a black circle in the *φ* = 0° phase) displacement is recorded for various applied voltages. Linear fit to the displacement data along with the goodness of the fit is represented

We perform an independent verification of the mode shapes using a laser Doppler vibrometer (LDV). LDV videos of the tangential mode and *n* = 2, 3 WGM are provided in the Supplementary material (Supplementary Videos 1,2, and 3 respectively). Thus, experimental values of resonant frequencies obtained by RF measurements are verified using LDV since the detected mode shapes and their frequency concur with the dips observed in RF data. A comparison of the resonant frequencies for different radii resonators is shown in Fig. 2b. For larger radii, the agreement between calculated and experimental data is greater due to the influence of the side tethers getting less significant compared to the resonant body size. During the LDV measurements, cartesian grids (as opposed to the polar distribution of data points) are employed for scanning the top surface of the resonators in order to establish equal spacing between data points and eliminate any possible spatial aliasing and geometrical biasing of the circular scanned space. Snapshots of the measured WGM are shown in Fig. 3a at different phases of one cycle of oscillation for a disk resonator with a radius of 60 μm at 8.42 MHz. LDV measurement and data acquisition technique is described in detail in the Supplementary information (Supplementary Note 2).

We record the *Y*-displacement at two locations on the disk for the *n* = 2 WGM. At Location-1, (denoted with a red circle) which is the edge of the disk we measured a displacement of 11.46 nm in the *Y*-direction for an excitation amplitude of 1 V as shown in Fig. 3b, which is a comparable value to that of electrostatically transduced BAW gyroscopes. Since the complete mapping of each disk resonator takes about 50 min, the LDV measurement grid may drift with respect to the resonator over multiple scans. Therefore, in order to measure linearity, we chose Location-2 (denoted with the black circle) which resides slightly towards the center to achieve a more stable measurement point as depicted in Fig. 3a. The measured *Y*-displacement at this location shows linear displacement with applied voltage amplitude for the WGM as shown in Fig. 3b. The inset shows the linear equation in *y* = *mx* + *n* form along with *R*^2^ value showing the goodness of the fit.

**Fig. 4:**
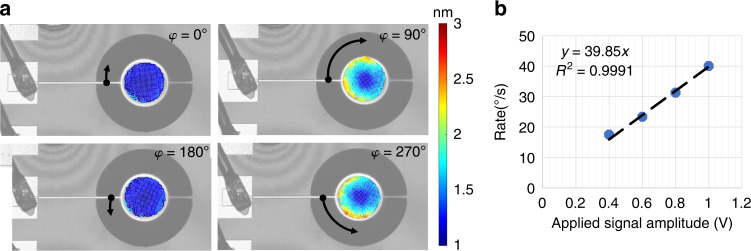
LDV results for tangential mode. **a** LDV snapshots of disk resonator with *r* = 60 μm radius shown at different phases of a single oscillation cycle at *φ* = 0°, *φ* = 90°, *φ* = 180°, and *φ* = 270°. **b** Rate of rotation is calculated from the *x*- and *y*-displacement, and plotted with respect to applied signal amplitude. Linear fit of the data points is presented in the inset

We also measured the tangential mode using LDV. Snapshots of a single oscillation cycle are provided in Fig. 4a for the same 60 μm radius device. Since LDV measures in-plane displacement in *X*- and *Y*-directions, we calculated the angular displacement along a radial line with an average displacement of 19 μm at 335 kHz, which is the resonant frequency of the tangential mode for a disk with *r* = 60 μm radius. This corresponds to a rotation rate of 40°/s when a 1 V amplitude excitation signal is applied. In Fig. 4b, the rate data is recorded for different voltage amplitude values showing a linear dependency of the rotation rate on the applied signal.

## Discussion

We achieved transverse actuation of tangential and compound mode family resonant shapes on a piezoelectric disk resonator using a self-aligned single-electrode exploiting the unique in-plane anisotropy of [011] oriented single crystal PMN–PT. We developed a wafer-scale fabrication procedure involving a combination of wet etching and ion milling of PMN–PT films that is thickness non-uniformity tolerant and capable of producing a vertical side-wall profile with dry etching technique. Direct excitation of tangential mode is attractive for a biological sample and viscosity sensing^23^ due to its isochoric features. This design makes it possible to take advantage of tangential surface displacement of disks with various radii values without the undesired out-of-plane displacement that is caused by misalignment^24^. Considering gyroscope applications of the *n* = 2 WGM, tip displacement of 11.46 nm is measured in LDV with 1 V amplitude actuation signal. The self-aligned feature of this actuator can be attractive for quadrature-error-free gyroscopes^25^, and tangential-mode TED-free oscillators. It is also important that obtained rotation rate values of the tangential mode and its linear dependency on the amplitude of the applied signal indicate that the tangential mode itself can serve as a micro rate table for in-site scale-factor calibration of gyroscopes^26–28^. Therefore, this technology provides the key building block for a BAW MEMS gyroscope with a built-in primary calibration stage.

## Materials and methods

### Device fabrication

**Fig. 5:**
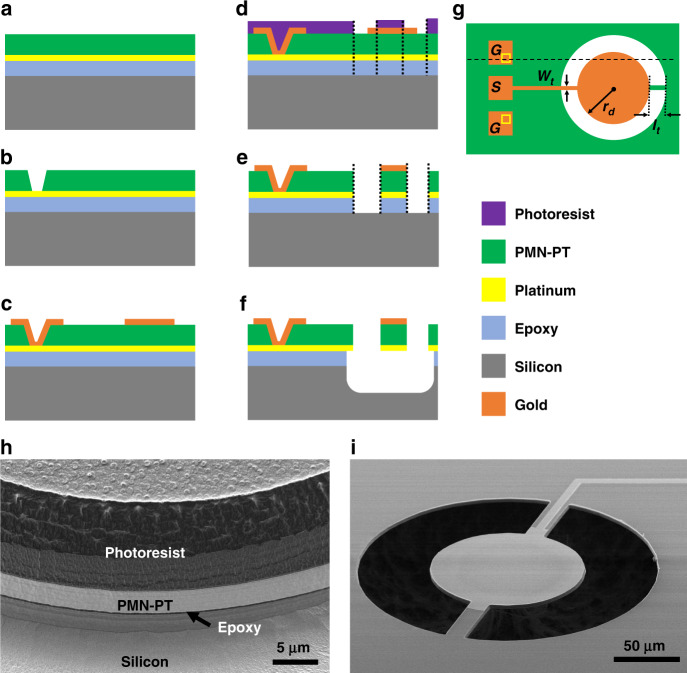
Fabrication process and SEM images. **a** Initial layer stack schematic representation. **b** Wet etching via openings for bottom electrode access. **c** Top metal deposition of Au layer. **d** Lithographic patterning of etch gaps defining the resonator body smaller than the top electrode ensuring self-alignment. **e** Ion milling (CHISEL) of resonator body along with side tethers. **f** Releasing the resonator by XeF_2_ etching of the silicon layer. **g** Schematic top view of the fabricated disk resonator and geometrical definitions. **h** SEM image of the sidewall after CHISEL etch with annotated layers. Due to the cyclic changing incident ion beam, the photoresist layer seems to have different textures on the sidewall. **i** SEM image of the suspended device with two side tethers

Single crystal [011]_c_ PMN-PT thin film samples (20 mm × 21 mm and ~6 μm-thick) are provided by iBule Photonics as attached to the silicon substrate by means of epoxy application between the film and the silicon substrate. While most bonding and fabrication process steps are identical to typical single-crystal Piezo-on-Insulator (POI) resonator fabrication, the most notable differences are depicted in Fig. 5d and e. The schematic cross-section of the initial layer stack is depicted in Fig. 5a. Fabrication of the resonators starts with wet etching of the PMN-PT film to get electrical access to the bottom electrode Pt layer as shown in Fig. 5b, where the Pt layer acts as a natural etch stop in a 10% HCl acid etch. The top electrode pattern along with the probe test pads and routing metal is deposited at the same lithographic step using liftoff. The top metal layer is evaporated as Ti/Au (10 nm/100 nm), where a 10 nm-thick Ti layer is used as the adhesion layer. In this step, the top electrode of the disk is defined slightly larger to ensure that even in the presence of misalignment of the resonator body to the top electrode, the entire resonator top surface is covered by the top metal electrode. The resonator body is defined along with the side tethers in the same lithographic step eliminating possible anchor-to-resonator body misalignment that would cause more spurious modes or more pronounced mechanical loss through anchors. The anisotropic etching of the resonator body is realized using ion milling until the silicon layer underneath the film is fully exposed. Different from conventional ion milling, a new ‘CHISEL’ (CHanging Incident beam-angle for Sidewall Etching and Lapping) approach is developed and described in detail (Supplementary Note 1). The etching step is divided into cycles of varying ion beam incident angles making it possible to define vertical and residue-free side walls as depicted in Fig. 5h with annotated layers. Finally, exposed silicon is sacrificially etched using XeF_2_ resulting in the self-aligned electrode resonator Fig. 5f. An SEM image of the released disk resonator is shown in Fig. 5i.

### Electrical and optical measurement setup

*S*_11_ measurements are performed using a ground-signal-ground (GSG) probe along with a network analyzer upon following a standard short open and load (SOL) calibration protocol on an appropriate calibration substrate. Samples are wire-bonded to a fan-out PCB that allows coaxial connector connectivity for excitation for LDV measurements, which are performed using Polytec MSA-100-3D. Surface modifications are implemented using photoresist patterns to enable lateral displacement detection with an enhanced diffuse scattering of the laser from the top electrode surface, which otherwise provided only specular reflection due to the smoothness of the top electrode surface. All RF and LDV measurements are performed at room temperature and atmospheric pressure. Setup schematic of both electrical and LDV measurements is provided in Supplementary Note 6.

## Supplementary information

Supplementary Information

Laser Doppler Vibrometry real-time video of tangential mode of vibration

Laser Doppler Vibrometry real-time video of n=2 wine-glass mode of vibration

Laser Doppler Vibrometry real-time video of n=3 wine-glass mode of vibration

## Data Availability

The data and the code used to produce the plots along with the raw images and video files within this work are available on Zenodo (10.5281/zenodo.7850137). All other data used in this study are available from the corresponding authors on reasonable request.
