# Supplementary material for: Self-aligned single-electrode actuation of tangential and wineglass modes using PMN-PT
Source: Microsyst Nanoeng. 2023 May 4;9:52. doi: 10.1038/s41378-023-00521-3 (PMC10160121; doi:10.1038/s41378-023-00521-3)
Supplement: Supplementary file 1 — Supplementary Information [file 41378_2023_521_MOESM1_ESM.docx]

Supplementary Information to: Self-Aligned Single-Electrode Actuation of Tangential and Wineglass Modes using PMN-PT

Ozan Erturk^1^, Kilian Shambaugh^2^, Ha-Seong Park^3^, Sang-Goo Lee^3^, Sunil A. Bhave^1^,^†^

*^1^OxideMEMS Lab, Purdue University, West Lafayette, IN, USA*

*^2^Polytec Inc. Irvine, CA, USA*

*^3^iBULe Photonics Company Ltd., Incheon 21999, South Korea*

*^†^E-mail:* [*bhave@purdue.edu*](mailto:bhave@purdue.edu)

**Supplementary Note 1: CHISEL Process**

CHanging Incident beam-angle for Sidewall Etching and Lapping (CHISEL) process is developed to realize a vertical and residue-free sidewall. We used AJA ATC-2036-IM Ion milling tool that is capable of operating with tilted sample stage with respect to the incident ion beam in range of *14°<θ <80°*. The etching angle *θ* is defined as the angle between the sample surface normal and the incident ion beam axis. Supplementary Figure 1a shows an SEM image of the photoresist pattern of a disk resonator with a side tether on PMN-PT. While vertical etching is performed at *θ=14°* to maximize the etch rate, non-volatile byproducts are re-deposited on the sidewall of the photoresist and the PMN-PT disk as shown in Supplementary Figure 1b. Changing the incident beam to *θ=80°* enables etching of the re-deposited material off of the sidewall as shown in Supplementary Figure 1c. This cycle is repeated until the PMN-PT around the disk is fully etched, and silicon layer underneath is fully exposed.

a

b


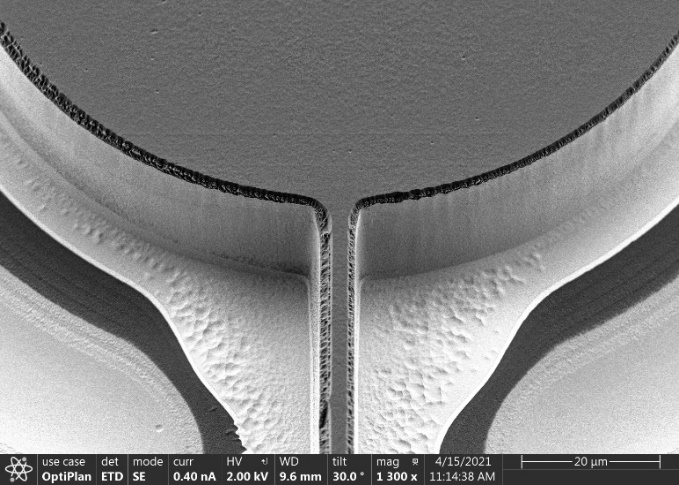


PMN-PT

Photoresist

20 μm

**
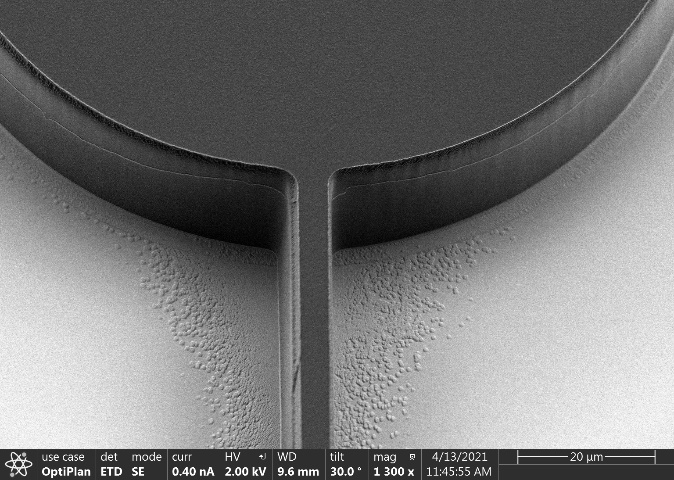
**

Photoresist

Epoxy

PMN-PT

20 μm

Si

c

Epoxy

PMN-PT


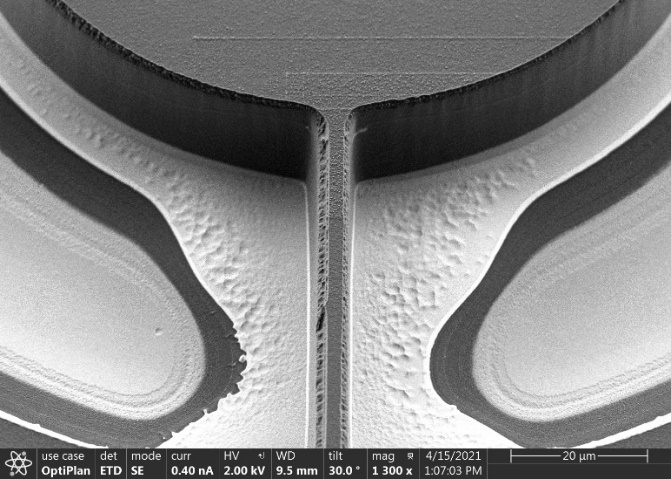


Photoresist

Si

20 μm

**Supplementary Figure 1: SEM images of CHISEL process showing the layers at different stages of etching. a** SEM image before vertical etching at *θ=14°*, where the photoresist sidewall is vertical and free of re-deposition. **b** SEM image after a cycle of vertical etching at *θ=14°*, where the photoresist sidewall is covered with re-deposited PMN-PT. Note that the vertical etching starts to exposes the silicon layer as well as the epoxy layer. **c** SEM image of the same device after shallow etching at *θ=80°*, where the re-deposited residues are cleared.

**Supplementary Note 2: LDV Measurement and Data Acquisition**

LDV measurements are performed using Polytec MSA 100-3D Mirco System Analyzer. Samples were wire-bonded to a fan-out PCB with an SMA connector that allowed electrical excitation through the internal signal generator of the MSA 100-3D system. Pyramid shaped photoresist patterns are defined before releasing the disks in order to enhance diffuse scatter of the LDV laser to increase the signal return for the in-plane displacement detectors. Supplementary Figure 2a shows the SEM image of a disk resonator with the photoresist patterns with a close-up SEM image of the patterns in Supplementary Figure 2b. A positive tone photoresist with a thickness of 1.5 µm is exposed with periodic circular patterns of 1.5 µm diameter and 3.0 µm pitch. Overexposure of the photoresist with circular patterns causes pyramid-like features as opposed to vertical sidewalls enabling diffuse scatter of the LDV laser.


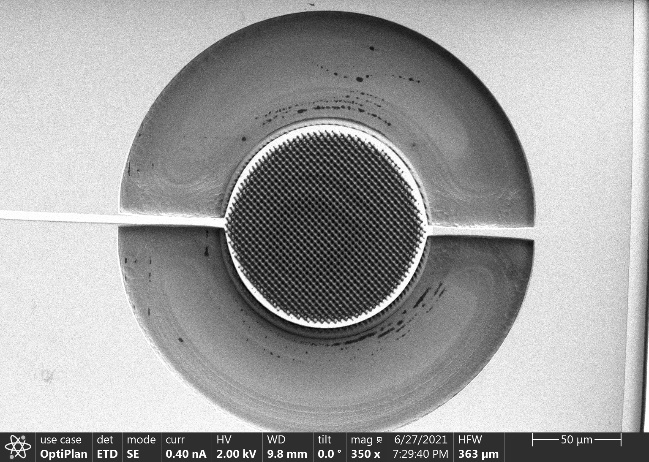

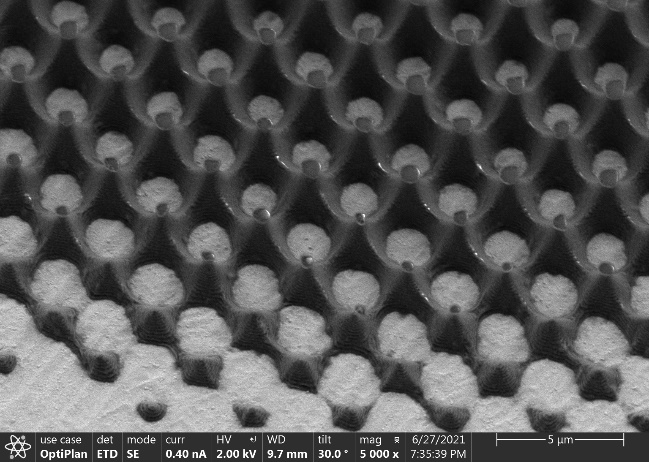


a

b

5 μm

50 μm

**Supplementary Figure 2: SEM images of the surface modifications on disk resonators to enhance the diffuse scatter of the LDV laser**. **a** SEM image of a disk resonator with photoresist patterns on top electrode. **b** Close-up SEM image of the pyramid-like photoresist patterns enhancing the diffuse scatter of the laser from the top surface. Note that the patterns fade out with decreasing thickness towards the edge of the disk due to photoresist thinning around the step-height difference.

LDV measures doppler shift of the probe laser after the laser beam interacts with the surface of interest. The reflected beam with doppler shift is decoded into velocity information in the x- and y-directions for each scan point. An array of scan points on the disk surface is defined using Cartesian grid in order to eliminate possible spatial aliasing and geometric biasing.

LDV measurement of the devices are performed in two stages. First, a periodic chirp signal is applied to measure the broadband frequency response of the device. Then resonance peaks with dominant in-plane components are identified. In the second stage of the measurement, same scan points are run with a single tone excitation and increased averaging to reduce the noise floor. Polytec PSV software calculates displacement and velocity in the frequency domain, and by taking advantage of complex averaging generates deflection shape videos by scaling the frame rate and exaggerating the deflection of each scan point (Supplementary Videos 1,2 and 3).

**Supplementary Note 3: Modified Butterworth-Van Dyke (mBVD) circuit model**

Piezoelectric resonator modelling is essential in design of devices in various applications. Transmission line modeling (also known as Mason Model) of Bulk Acoustic Wave (BAW) or contour mode resonators is a prevalent approach for modeling such devices that predict the broadband response. Lumped element circuit modeling of such devices, on the other hand, is more effective in system level analysis due to ease of integrating with peripheral electronics or optimization of specific resonant modes. A simple 1-port resonator can be represented using series R-L-C components in parallel with a plate capacitance C_0_ that represents the parallel plate capacitance of the piezoelectric layer sandwiched with electrodes as shown in Supplementary Figure 3a below [1]. Modified model, presented in Supplementary Figure 3b, includes series resistance (R_s_) to emulate the electrical losses, while another resistance between the parallel plates of the piezoelectric material is added to model the relevant material losses [2].


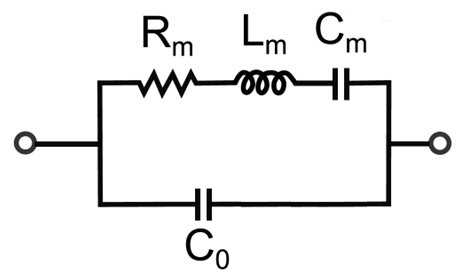

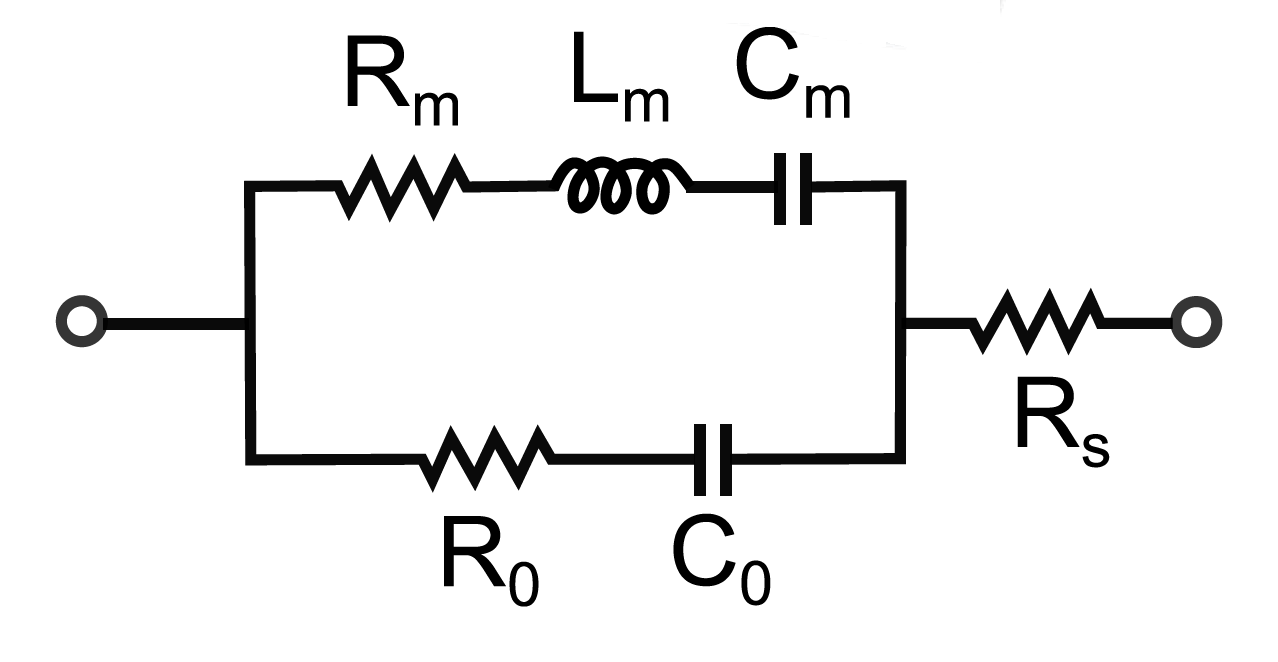


(b)

(a)

**Supplementary Figure 3: Lumped element model of a piezoelectric resonator**. **a** Butterworth-Van Dyke (BVD) model of a piezoelectric resonator with motional elements (*R_m_, L_m_*, and *C_m_*) corresponding to the mechanical resonance at a particular frequency while parallel shunt capacitance C_0_ models the static plate capacitance of the piezoelectric material determined by the electrode area and distance. **B** Modified Butterworth-Van Dyke (mBVD) model adds a series resistance *R_s_* to the overall model to include electrical losses, while another resistance (*R_0_*) in series with the plate capacitance is used to model the material losses.

**Supplementary Note 4: Alternative S_11_ plots**

Below are the S_11_ plots as a function of radius reproduced in alternate formats. Supplementary Figure 4a shows the plot with marked points for n=2 and n=3 wine-glass mode shapes while in Supplementary Figure 4b, the same plot presented in the manuscript is plotted with Y-offset.

(b)

(a)


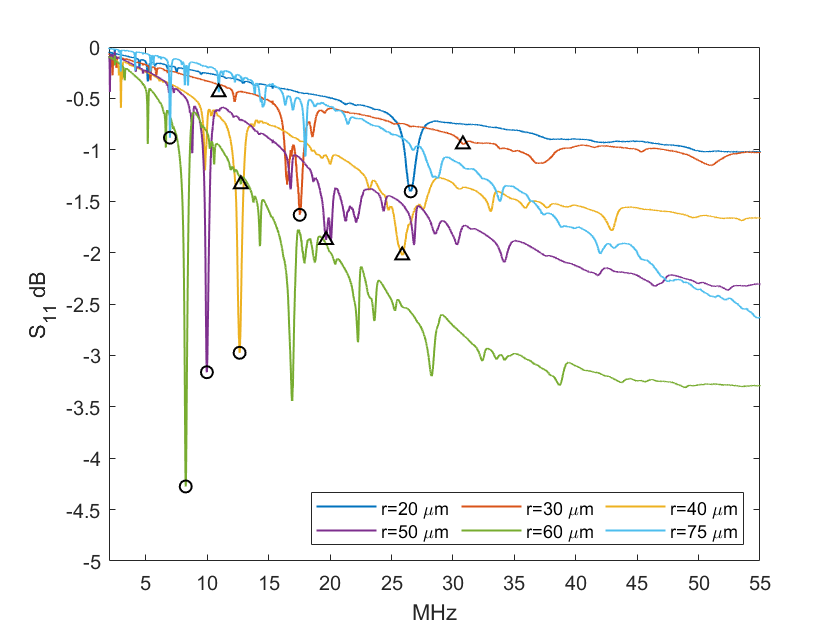

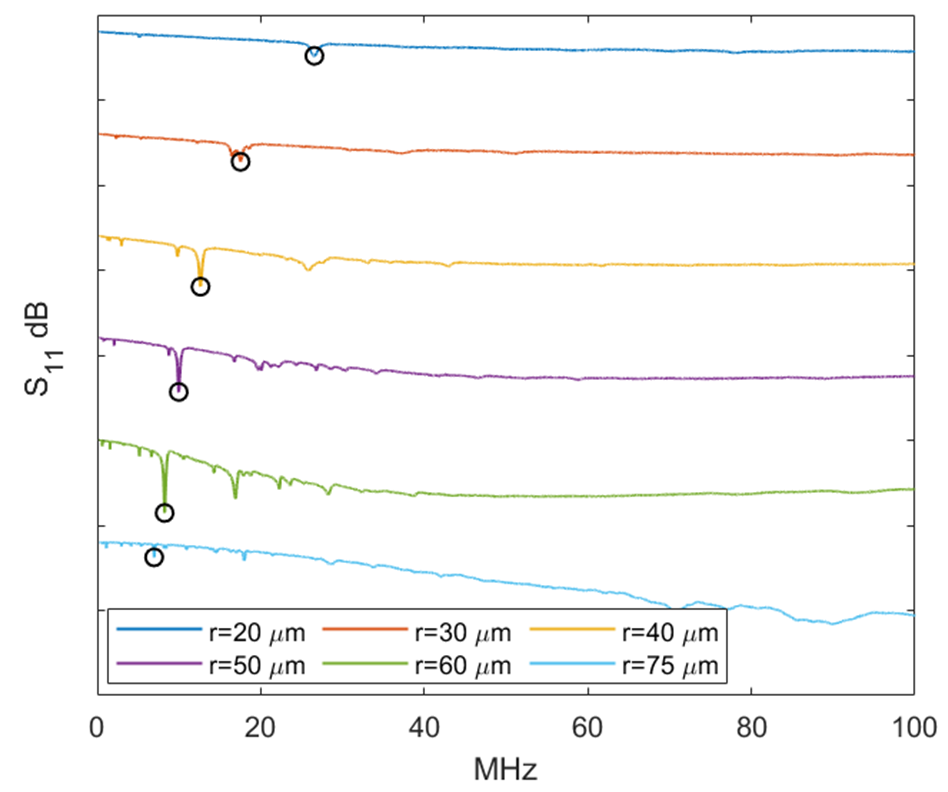


(a.u.)

**Supplementary Figure 4: Alternative S_11_ plots of the devices with different radii**. **a** Overlaid S11 data with different radii where circle markings indicate the n=2 wine-glass mode and triangle markings show n=3 wine-glass mode resonance. **b** S_11_ plot of the same data represented in Figure 2a in the manuscript with Y-offset.

**Supplementary Note 5: Resonant behavior of r=30 µm around WGM frequency**

The S_11_ plot corresponding to the device with radius of 30 µm shows another dip in the close vicinity of the wine-glass mode resonant shape as can be seen in Figure 2a. Two modes with similar quality factors are exhibited in this plot and their mode shape is shown using the FEM simulation tool. Supplementary Figure 5 below shows the eigenmode simulations depicting the exaggerated deformation along with color map of total displacement. It is seen that n=2 wine-glass mode shape has a resonant frequency of 16.893 MHz while the other depicted out-of-plane mode has a frequency of 16.339 MHz with very similar imaginary component magnitude. Since two modes are scaled differently with radius, these two modes are not as closely spaced for disk resonators with other radius values.

**
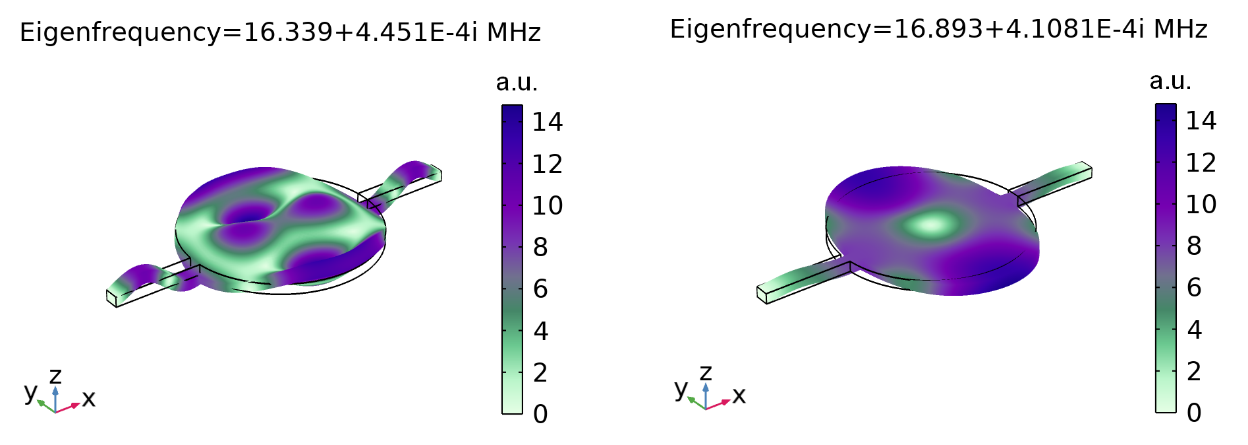
**

(b)

(a)

**Supplementary Figure 5:** **a** Eigenmode simulation result of an out-of-plane mode with resonant frequency of 16.339 MHz **b** Eigenmode simulation result of the n=2 WGM with resonant frequency of 16.893 MHz Note that the two modes are in closely spaced in frequency as the RF reflection measurement result shows.

**Supplementary Note 6: Experimental setup schematic**

RF reflection measurements are performed at room temperature and atmospheric pressure using a network analyzer and a probe station as depicted in Supplementary Figure 6a. Formfactor ACP 40 GSG probes are used to contact the PMN-PT chip for probe tests after 1-port Short-Open-Load (SOL) calibration.

Laser Doppler Vibrometer (LDV) measurements are performed using Polytec MSA-100-3D system. Since there was no RF probe integration to the system, a fan-out PCB is used to connect the disk resonators to the electrical excitation of the MSA-100-3D system via an SMA connector on the PCB as shown in Supplementary Figure 6b. The electrical excitation of the disk resonators is then provided by the synchronized RF source on the MSA-100-3D device as shown in the figure below.


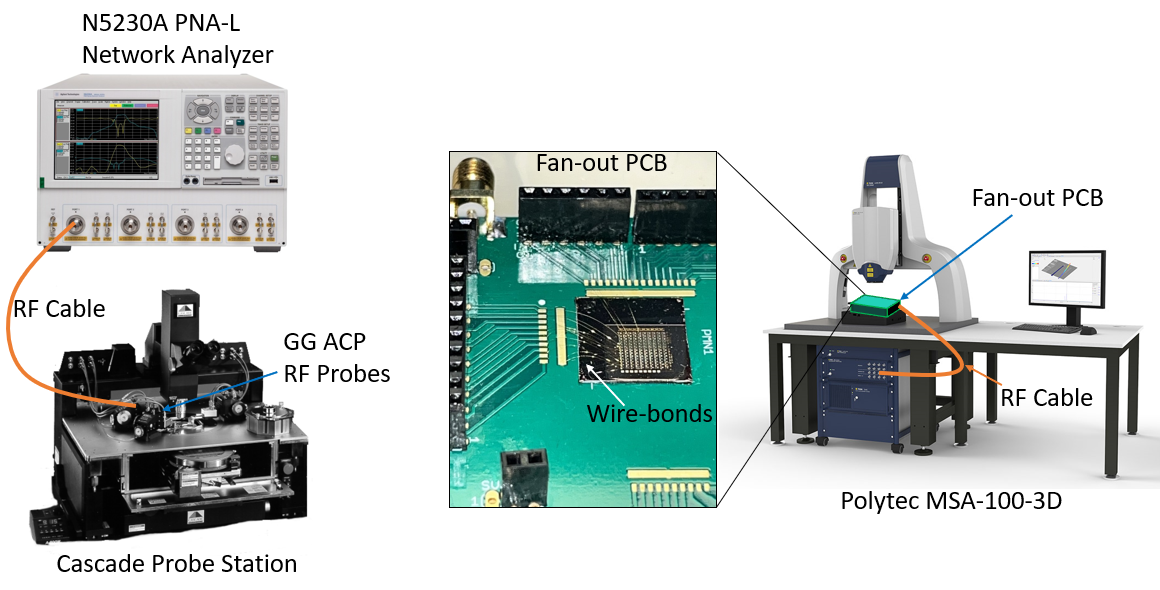


(a)

(b)

**Supplementary Figure 6: Experimental setup schematic representation.** **a** RF reflection measurement setup with a network analyzer and probe station, where electrical connection to the chip is provided with ACP 40-GSG RF probes. **b** LDV measurement schematic representation along with zoomed in picture of the fan-out PCB with PMN-PT chip wire-bonded for electrical connection to the LDV system.

**References:**

[1] “IRE Standards on Piezoelectric Crystals-The Piezoelectric Vibrator: Definitions and Methods of Measurement, 1957,” *Proc. IRE*, vol. 45, no. 3, pp. 353–358, Mar. 1957, doi: 10.1109/JRPROC.1957.278371.

[2] J. D. Larson, P. D. Bradley, S. Wartenberg, and R. C. Ruby, “Modified Butterworth-Van Dyke circuit for FBAR resonators and automated measurement system,” in *2000 IEEE Ultrasonics Symposium. Proceedings. An International Symposium (Cat. No.00CH37121)*, Mar. 2000, vol. 45, no. 3, pp. 863–868 vol.1, doi: 10.1109/ULTSYM.2000.922679.
